# Adrenal Insufficiency as a Contributor to Severe Hypoglycemia in Late Dumping Syndrome: A Case Series

**DOI:** 10.7759/cureus.75195

**Published:** 2024-12-06

**Authors:** Ken Kanazawa, Takeki Ito, Mai HIjikata, Koichiro Kuwabara

**Affiliations:** 1 Department of Diabetes, Metabolism and Endocrinology, Japan Labor Health and Safety Organization, Tokyo Rosai Hospital, Tokyo, JPN

**Keywords:** adrenal insufficiency, late dumping syndrome, reactive hypoglycemia, severe hypoglycemia, the elderly

## Abstract

Severe hypoglycemia (SH) is a significant risk, particularly in the elderly, and adrenal insufficiency (AI) may be a contributing factor. This study examines six cases of late dumping syndrome (LDS)-induced reactive hypoglycemia (RH), with AI as a potential trigger. Three of the six patients were diagnosed with AI, and one experienced a hypoglycemic coma. A significant correlation between blood glucose and insulin levels was found in the AI group. These findings suggest the potential role of AI in the development of SH in LDS patients after gastrectomy. Further research is needed for confirmation.

## Introduction

Severe hypoglycemia (SH) is a significant prognostic factor, especially in the elderly, as it can lead to cognitive and mood disorders, coma, fall-related injuries, cardiovascular events, and arrhythmias [1-5]. While SH is primarily caused by iatrogenic hypoglycemia due to hypoglycemic drugs in patients with diabetes, idiopathic cases such as adrenal insufficiency (AI) must also be considered [6,7]. This study reports six cases of reactive hypoglycemia (RH), including SH, associated with late dumping syndrome (LDS) after gastrectomy, where AI was suspected as a trigger.

## Materials and methods

Study design and ethical considerations

This case series was conducted at the Department of Diabetes, Metabolism, and Endocrinology, Tokyo Rosai Hospital, Japan, from April 2015 to February 2024. Informed consent was obtained orally, and participants were given the option to opt out of the study. Eligibility assessments were conducted and reviewed in accordance with the Declaration of Helsinki. The study protocol was approved by the Institutional Review Board of Tokyo Rosai Hospital (REC No. 06-17).

Study participants

Participants were at least 18 years old, post-gastrectomy, and with episodes of hypoglycemia (e.g., hypoglycemic coma, nausea, dizziness, cold sweats) and were treated under close observation. Each patient was evaluated for LDS with a 75 g oral glucose tolerance test (75 g OGTT) and for AI with an endocrine stress test. Exclusions included recent myocardial infarction, current malignancy, and acute infection. Furthermore, other factors contributing to SH included excessive alcohol consumption, severe malnutrition, severe liver disease, dialysis, type 1 diabetes, and the use of hypoglycemic drugs. Additionally, conditions such as insulinoma, autoimmune insulin hypoglycemia, and hormone deficiencies were excluded. The study did not include any exogenous steroid preparations (oral, ointment, inhalation, etc.).

75 g OGTT and reactive hypoglycemia

The 75 g OGTT involved an overnight fast, followed by ingestion of a 75 g glucose solution, with blood glucose (BG) and immunoreactive insulin (IRI) measured at 30-minute intervals for 180 minutes. RH was diagnosed if hypoglycemia occurred during the late phase (120-180 minutes), indicating LDS [8].

AI and endocrine stress test

AI was suspected if early morning blood cortisol was <16 μg/dL. Definitive diagnosis is made using a standard dose corticotropin stimulation test (SDST), low dose corticotropin stimulation test (LDST), and corticotropin-releasing hormone stimulation test (CRHST). AI was diagnosed if peak cortisol levels were <18 μg/dL (SDST/LDST or CRHST) [9,10]. Serum cortisol and plasma adrenocorticotropic hormone (ACTH) concentrations were measured using electrochemiluminescence immunoassay on a Cobas 8000 system (Roche Diagnostics, Basel, Switzerland). The established reference ranges were 7.1-19.6 μg/dL for serum cortisol and 7.2-63.3 pg/mL for plasma ACTH.

Statistical analysis

Categorical variables were reported as raw frequencies (%), and continuous variables were presented as mean ± SD or median (interquartile range). Simple linear regression was used to assess the relationship between BG and IRI in the 75 g OGTT, with significance tested for the regression coefficient (β1) and model fit assessed using R². All statistical analyses were performed using JMP version 12 (SAS Institute Inc., Cary, NC). P-values <0.05 were considered statistically significant.

## Results

Case 1

**Figure 1:**
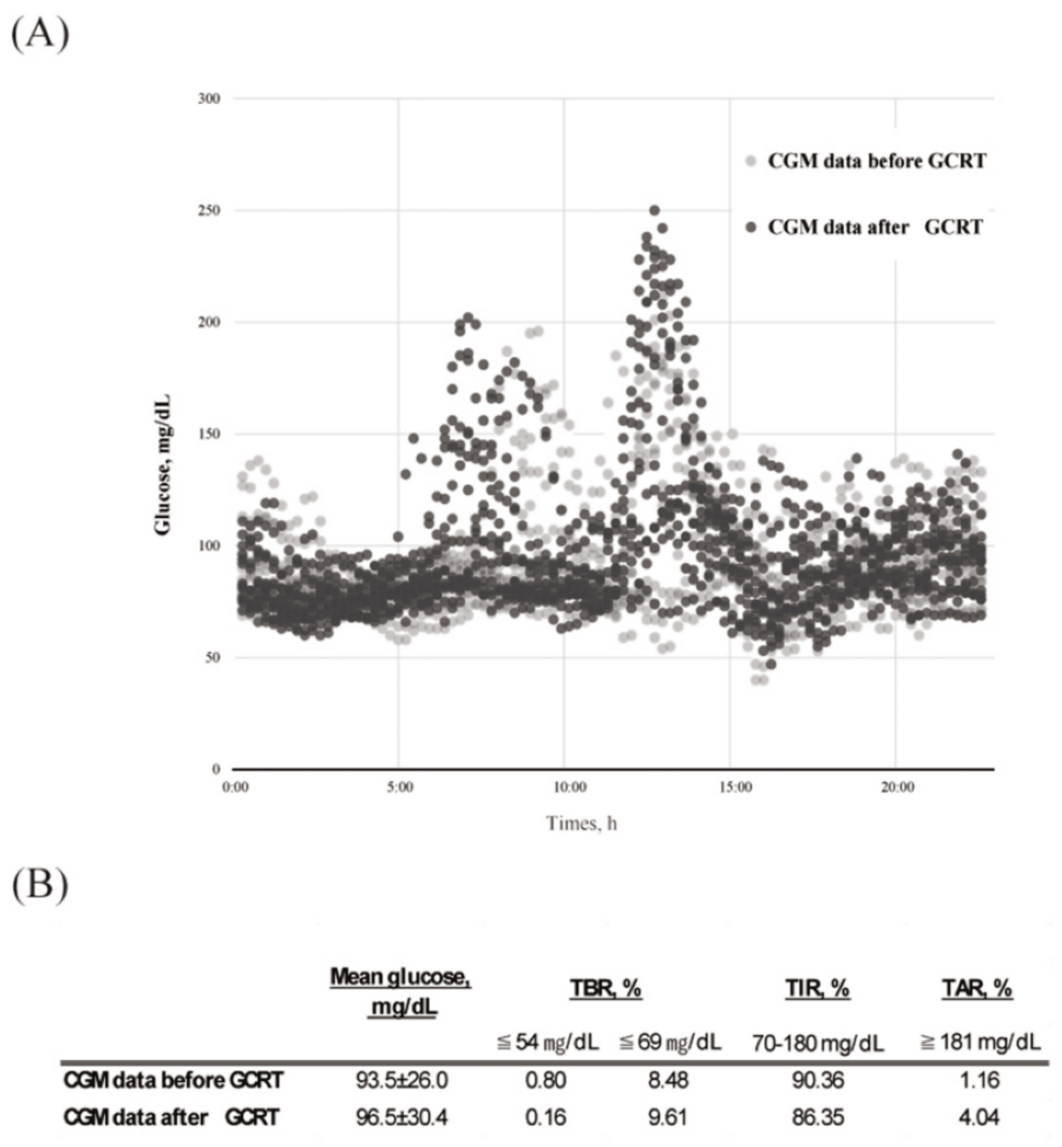
Comparison of CGM data before and after GCRT in Case 1 A: CGM data before and after GCRT; B: Mean glucose and time in range in CGM before and after GCRT After GCRT, the incidence of severe hypoglycemia (glucose ≦ 54 mg/dL) decreased. Abbreviations: CGM, continuous glucose monitoring; GCRT, glucocorticoid replacement therapy; h, hour; TBR, time below range; TIR, time in range; TAR, time above range

K.S., a 66-year-old male who underwent gastric jejunal bypass surgery for gastrinoma three years ago, presented with current postprandial weakness, numbness, and cold sweats, alleviated by carbohydrate intake. His medical history showed no endocrine disorders, such as insulinoma, use of hypoglycemic drugs, and heavy alcohol consumption. His medications included esomeprazole (10 mg). Initial tests showed normal glucose levels, but continuous glucose monitoring (CGM) revealed SH (Figure 1). A 75 g OGTT confirmed late-phase hypoglycemia, leading to a diagnosis of LDH-induced RH. Further tests showed low cortisol and ACTH (9.0 μg/dL and 14.0 pg/mL), LDST (peak cortisol: 18.4 μg/dL), and CRHST (peak cortisol: 13 μg/dL), diagnosing AI. We initiated glucocorticoid replacement therapy (GCRT) to prevent SH. After GCRT, the long-standing distracting symptoms disappeared, and CGM showed a reduction in SH (Figure 1).

Case 2

N.H. is an 86-year-old man who underwent a subtotal gastrectomy with Roux-en-Y gastric bypass (RYGB) reconstruction for gastric cancer seven years ago. His medical history included hypertension and alcoholic liver disease, though his alcohol consumption was low. No significant findings were associated with his current medications. Recently, before dinner, he developed generalized convulsions and was transported to the hospital in a coma. He was diagnosed with SH (blood glucose: 36 mg/dL), and at that time, his basal cortisol level was relatively low (11.4 μg/dL; LDST (peak cortisol): 15.1 μg/dL), diagnosing AI. In this case, AI is thought to have contributed to SH induced by LDS.

Case 3

H.S. is an 82-year-old man who underwent a distal gastrectomy with Billroth I reconstruction for gastric cancer in 1983. His medical history includes hypertension, chronic renal failure, and Hashimoto's thyroiditis. His only medications are levothyroxine sodium (25 μg) and acarbose (100 mg). In the past, he was diagnosed with LDS-induced RH, and he has been managing it for many years through dietary adjustments and acarbose. However, he continued to experience dizziness, particularly after eating, and it did not improve. Further tests revealed low cortisol and ACTH levels (4.4 μg/dL and 22.4 pg/mL, respectively), LDST (peak cortisol: 14.3 μg/dL), and a CRHST (peak cortisol: 14.6 μg/dL), leading to a diagnosis of AI. In this case, AI was thought to be involved in LDS-induced RH. After GCRT, the long-standing dizziness resolved.

Case 4

S.U., is a 65-year-old female who underwent a distal gastrectomy with Billroth I reconstruction for gastric cancer in 1968, presented with preprandial hypoglycemia. Her medical history includes hypertension, chronic obstructive pulmonary disease (COPD), and sleep apnea syndrome (SAS). No significant findings were associated with her current medications. Her 75 g OGTT showed particularly pronounced delayed hyperinsulinemia among the six cases, and prolonged fasting between meals was suggested to contribute to hypoglycemia. The basal cortisol level was low (7.1 μg/dL), suggesting the involvement of AI, but this was ruled out by endocrine stress testing.

Case 5

H.S. is an 80-year-old man who underwent a distal gastrectomy with Billroth I reconstruction for gastric cancer 10 years ago. His medical history includes dementia and hypertension. He is taking donepezil (5 mg), carvedilol (2.5 mg), and amlodipine (2.5 mg) orally. Recently, after consuming alcohol following dinner, he experienced generalized convulsions and was transported to the hospital in a coma. He was found to have SH (blood glucose level: 31 mg/dL). Subsequently, intravenous glucose was administered in the emergency room, normalizing his blood glucose level and improving his consciousness. Although 75 g OGTT observed LDH-induced RH, AI was ruled out through endocrine stress testing. In this case, chronic kidney disease, alcoholic liver damage, and alcohol consumption are thought to have contributed to SH induced by LDS.

Case 6

S.U., a 63-year-old man who underwent a subtotal gastrectomy with RYGB reconstruction for gastric cancer 10 years ago, presented with preprandial hypoglycemic symptoms that were alleviated by carbohydrate intake. His medical history showed no endocrine disorders such as insulinoma, use of hypoglycemic drugs, and heavy alcohol consumption. As in Case 4, although the 75 g OGTT revealed LDH-induced RH, the basal cortisol level was low (9.7 μg/dL), suggesting the involvement of AI. However, this was ruled out through endocrine stress testing. The hypoglycemic symptoms were treated with snacks between meals, resulting in observed improvement.

Clinical characteristics of patients diagnosed with AI

Among the six patients with LDS-induced RH, three were diagnosed with AI through endocrine stress testing (Tables 1-2). In the AI group, one patient experienced a hypoglycemic coma. None had total gastrectomy; surgical histories included gastric jejunal anastomosis, subtotal gastrectomy, and distal gastrectomy. The laboratory data for the AI group showed eosinophils of 9.5±8.5%, eGFR of 61.4±9.1 mL/min/1.73m², Na of 137.3±5.8 mEq/L, K of 4.3±0.6 mEq/L, FPG of 93.3±10.4 mg/dL, HbA1c of 5.7±0.7%, IRI of 6.6±3.6 μU/mL, homeostasis model assessment of insulin resistance (HOMA-R) of 1.6±1.0, and homeostasis model assessment of β-cell function (HOMA-β) of 77.3±27.9%. Endocrinological evaluation in the AI group revealed basal cortisol of 8.3±3.6 μg/L, ACTH of 48.5±52.6 pg/mL, LDST (peak cortisol) of 15.9±2.2 μg/dL, SDST (peak cortisol) of 19.0±2.5 μg/dL, CRHST (peak cortisol) of 13.8±1.1 μg/dL, and peak ACTH (CRHST) of 35.7±14.2 pg/mL.

**Table 1:** Comparison of the patients' baseline characteristics This table compares the six cases in this study, classified as adrenal insufficiency or none. Continuous variables are expressed as mean ± standard deviation (SD). Abbreviations: M, male; F, female; BMI, body mass index; s/g, subtotal gastrectomy; d/g, distal gastrectomy; RYGB, Roux-en-Y gastric bypass; BI, Billroth-I reconstruction; WBC, white blood cell; Eosino, eosinophil; eGFR, estimated glomerular filtration rate; FPG, fasting plasma glucose; IRI, immunoreactive insulin

Case	Age, years	Sex	BMI, kg/m^2^	Episodes of hypoglycemia	Date of surgery	Surgical technique	Reconstructions	Alchool use	WBC, 10^2^ μL	Eosino, %	eGFR, mL/min/1.73 m^2^	Na, mEq/L	K, mEq/L	FPG, mg/dL	HbA1c, %	IRI, μU/mL	HOMA-R	HOMA-β, %
Adrenal Insufficiency																		
1	66	M	25.2	Sweating after eating	2015	Gastric jejunostomy		Yes	52	0.8	55.8	144	4.1	105	5.7	10.5	2.7	90
2	86	M	22.6	Loss of consciousness	2017	s/g	RYGB	Yes	39	9.9	71.9	134	4.9	85	5	5.9	1.2	96.6
3	82	M	19.3	Dizziness	1983	d/g	BⅠ	No	73	17.8	56.6	134	3.8	90	6.3	3.4	0.8	45.3
Mean ±SD	78 ±10.6		22.4 ±3.0						54.7 ±17.2	9.5 ±8.5	61.4 ±9.1	137.3 ±5.8	4.3 ±0.6	93.3 ±10.4	5.7 ±0.7	6.6 ±3.6	1.6 ±1.0	77.3 ±27.9
Not Adrenal Insufficiency																		
4	65	F	22.2	Preprandial hypoglycemia	1968	d/g	BⅠ	No	81	0.9	48.6	143	3.9	89	5.3	6.5	1.4	90
5	80	M	20.5	Loss of consciousness	2013	d/g	BⅠ	No	50	5.6	28.6	145	4.3	87	5.3	3.9	0.8	58.5
6	63	M	25.3	Preprandial hypoglycemia	2005	s/g	RYGB	No	68	2	113.6	138	4	105	5.7	8.4	2.2	72
Mean ±SD	69.3 ±9.3		22.7 ±2.5						66.3 ±15.6	2.8 ±2.5	63.6 ±44.4	142.0 ±3.6	4.1 ±0.2	93.7 ±9.9	5.4 ±0.2	6.3 ±2.3	1.5 ±0.7	73.5 ±15.8

**Table 2:** Endocrinological basal values and stress test results This table presents the endocrinological basal values and stress test results for the six patients in this study. Continuous variables are expressed as mean ± standard deviation (SD). Abbreviations: BC, basal cortisol; P-C, peak cortisol; LDSH, low dose corticotropin stimulation test; SDST, standard dose corticotropin stimulation test; CRHST, corticotropin releasing hormone stimulation test; P-ACTH, peak ACTH

Case	BC, μg/dL	ACTH, μg/dL	TSH, μIU/mL)	FT4, ng/dL	P-C (LDST), μg/dL	P-C (SDST), μg/dL	P-C (CRHST), μg/dL	P-ACTH (CRHST), μg/dL
Adrenal Insufficiency								
1	9	14	1.5	1.1	18.4	21.8	13	25.6
2	11.4	109	1.6	1.1	15.1	18	N/D	N/D
3	4.4	22.4	5.2	0.8	14.3	17.1	14.6	45.7
Mean±SD	8.3±3.6	48.5±52.6	2.8±2.1	1.0±0.2	15.9±2.2	19.0±2.5	13.8±1.1	35.7±14.2
Not Adrenal Insufficiency								
4	7.1	16.5	2.3	1.0	19.8	22	18.8	23.2
5	13.9	47.6	2.5	1.2	N/D	21.8	N/D	N/D
6	9.7	16.2	3.1	1.1	19.1	25.4	18	51.2
Mean±SD	10.2±3.4	23.4±21.5	2.6±0.4	1.1±0.1	19.5±0.5	23.1±2.0	18.4±0.6	37.2±17.2

**Figure 2:**
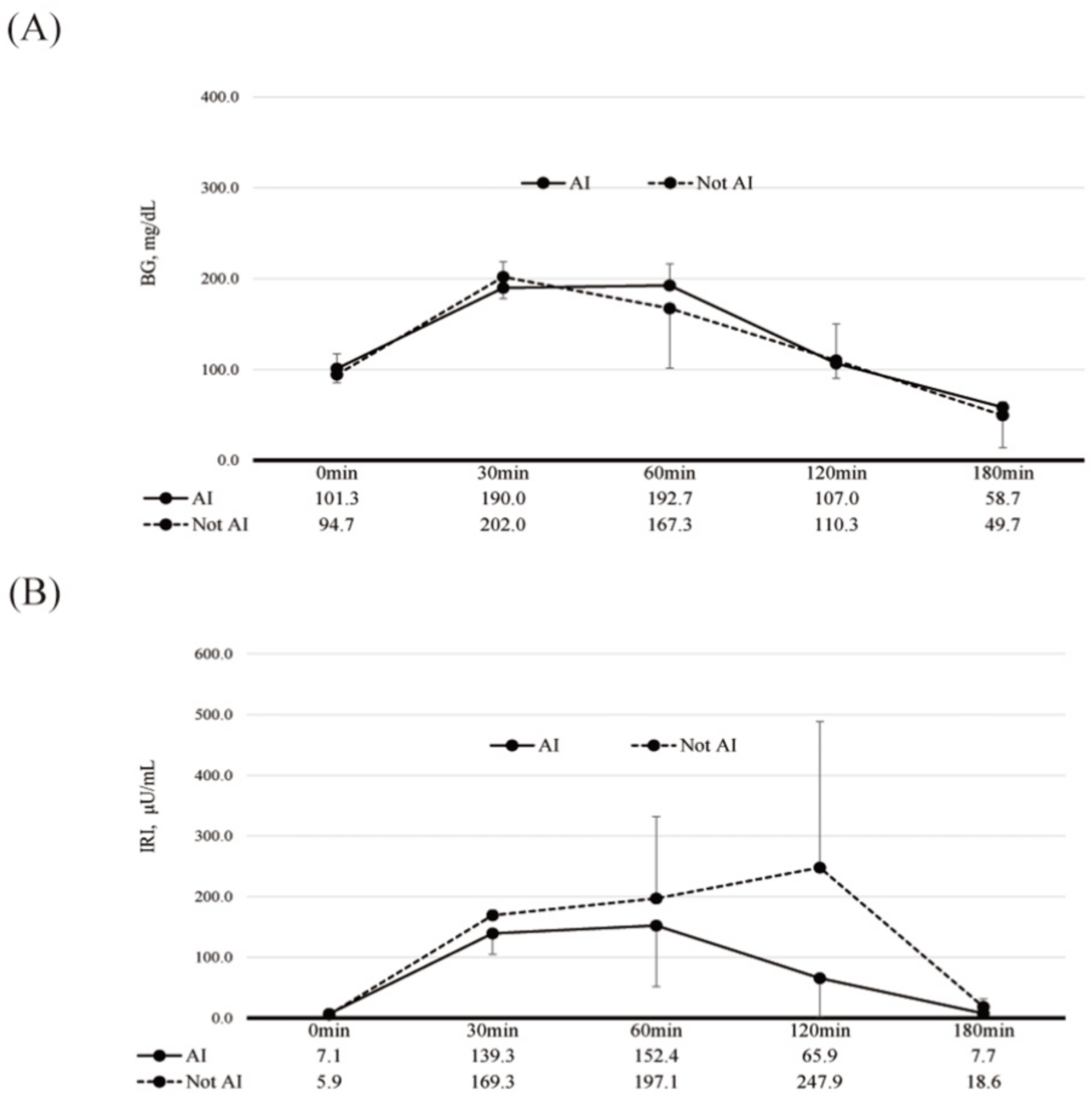
75 g OGTT results compared AI and not AI groups A: BG trends in 75g OGTT compared AI to Not AI groups; B: IRI trends in 75g OGTT compared AI to Not AI groups The figure shows BG and IRI trends in 75 g OGTT. No significant differences were found in BG and IRI between the groups. BG was the lowest at 180 minutes post-loading, while IRI peaked at 60 and 120 minutes and then sharply declined at 180 minutes. Abbreviations: 75 g OGTT, 75 g oral glucose tolerance test; AI, Adrenal Insufficiency; BG, blood glucose; IRI, immunoreactive insulin

**Figure 3:**
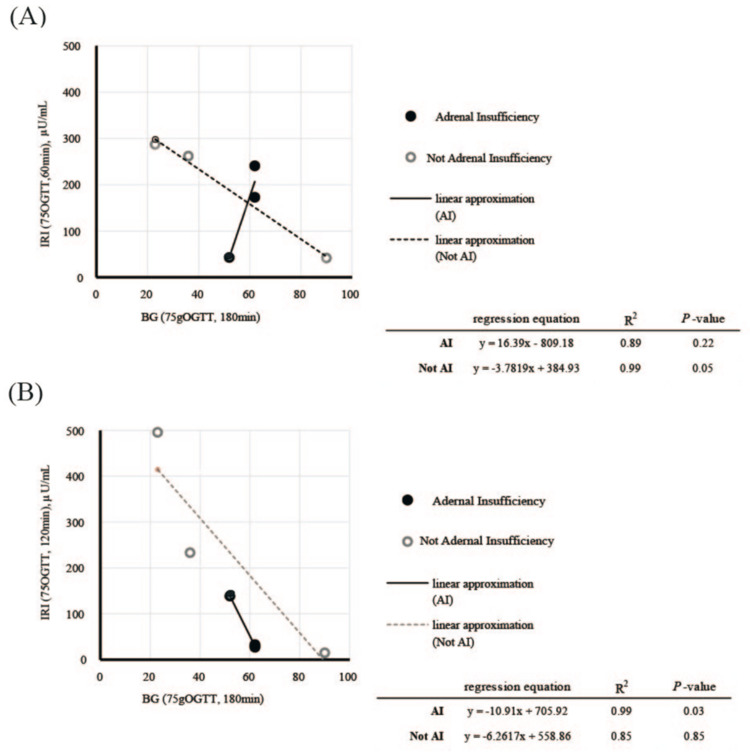
The relationship between BG and IRI in 75 g OGTT A: The relationship between BG at 180 minutes and IRI at 60 minutes; B: The relationship between BG at 180 minutes and IRI at 120 minutes. The figure shows the relationship between BG at 180 minutes and IRI at 60 and 120 minutes. In the AI group, a significant correlation was found between BG at 180 minutes and IRI at 120 minutes (β1 = -10.91, R² = 0.9982, P < 0.001). Abbreviations: BG, blood glucose; IRI, immunoreactive insulin; 75 g OGTT, 75 g oral glucose tolerance test; AI, adrenal insufficiency

In 75 g OGTT, no significant differences were found in BG and IRI levels between the AI and non-AI groups (Figure 2). In the AI group, on the other hand, a significant correlation was found between BG at 180 minutes and IRI at 120 minutes (β1 = -10.91, R² = 0.9982, P < 0.001) (Figure 3).

## Discussion

In this case series, we examined six patients who developed LDS-induced RH after gastrectomy, with three diagnosed with AI. Our findings suggest that impaired cortisol secretion, indicative of AI, may significantly contribute to SH in these patients. The 75 g OGTT revealed a significant correlation between IRI (120 min) and BG (180 min) in patients with AI, highlighting the importance of accurate diagnosis for better prognosis, especially since AI-related hypoglycemia can lead to severe outcomes such as coma.

This study provides new insight into the comorbidity of AI in patients with LDS-induced RH, a condition traditionally considered rare after pyloric gastrectomy or RYGB [8,11,12]. Our comprehensive endocrine stress testing revealed AI as a potential contributing factor to SH in LDS. Notably, our analysis showed that AI can cause hypoglycemia with lower insulin levels compared to patients without AI, emphasizing the need to consider AI in the management of RH.

Previous studies have identified LDS and islet cell hyperplasia or nesidioblastosis as primary causes of postprandial hypoglycemia following gastrectomy [8]. LDS alters the normal continuity of the upper gastrointestinal tract, leading to rapid glucose absorption in the jejunum and subsequent hyperinsulinemia, causing RH [13]. Meanwhile, hyperinsulinemic hypoglycemia due to islet cell hyperplasia or nesidioblastosis has been reported after RYGB [14,15]. In this study, we observed a rapid rise in BG at 30 and 60 minutes, followed by delayed hyperinsulinemia (at 60, 120, and 180 minutes) and eventual hypoglycemia (at 180 minutes) after a 75 g OGTT. This pattern suggests hyperinsulinemic hypoglycemia regardless of AI status. However, a significant correlation between BG at 180 minutes and IRI at 120 minutes was found only in AI, indicating that AI may exacerbate the severity of hypoglycemia after gastrectomy. Similar to this study, Uryu et al. showed the possible involvement of AI in LDS and hypoglycemia [16]. When AI appears several years after surgery, an aggressive endocrine stress test is recommended, taking into account possible complications of AI. Our findings suggest the need for more extensive endocrine testing in patients presenting with hypoglycemia after gastrectomy (mean 19 years) and are consistent with the evidence of their previous studies. Additionally, we found instances of SH in patients without AI, such as in cases where preexisting conditions (e.g., end-stage renal failure) may have played a role, highlighting the need for a comprehensive evaluation of multiple factors [17].

LDS-induced RH has traditionally been managed with dietary adjustments and pharmacotherapy, targeting rapid gastric emptying [8]. However, this study identifies AI as a contributing factor in some patients, suggesting that GCRT may be beneficial alongside standard treatments. Therefore, clinicians should consider evaluating adrenal function in cases of unexplained SH to improve diagnosis and patient outcomes.

Study limitations

This study has several limitations. The small sample size and the exclusion criteria described in the Methods section could have reduced interference with hypoglycemia but may have limited the applicability of the study results. Generalization of the findings will require further attention. Furthermore, the absence of a control group of post-gastrectomy patients without hypoglycemic episodes makes it difficult to assess the prevalence of AI. Additionally, further prospective studies are necessary to clarify the mechanisms linking hypoglycemia and AI in LDS patients.

## Conclusions

This study highlights the potential role of AI in the development of SH in LDS patients after gastrectomy. Clinicians should include AI in the differential diagnosis when managing LDS patients with SH and conduct appropriate endocrine evaluations. Further research is needed to refine management strategies for this complex interplay between endocrine dysfunction and hypoglycemia.
